# Platinum-resistant ovarian cancer: From mechanisms to treatment strategies

**DOI:** 10.1016/j.gendis.2025.101801

**Published:** 2025-08-11

**Authors:** He Li, Jia-Jia Sheng, Sheng-An Zheng, Po-Wu Liu, Nayiyuan Wu, Wen-Jing Zeng, Ying-Hua Li, Jing Wang

**Affiliations:** a The Affiliated Cancer Hospital of Xiangya School of Medicine, Central South University/Hunan Cancer Hospital, Changsha, Hunan 410013, China; b Hunan Provincial Key Laboratory of the Research and Development of Novel Pharmaceutical Preparations, the “Double-First Class” Application Characteristic Discipline of Hunan Province (Pharmaceutical Science), Changsha Medical University, Hunan 410219, China; c College of Pharmacy, Dali University, Dali, Yunnan 671000, China; d Department of Pharmacy, Xiangya Hospital, Central South University, Changsha, Hunan 410008, China

**Keywords:** Clinical trials, Mechanisms, Ovarian cancer, Platinum resistance, Prognosis-free survival

## Abstract

Over the last few decades, platinum-based chemotherapy has served as the standard chemotherapy in treating ovarian cancer (OC). While most patients initially respond well to platinum-based chemotherapy, approximately 70% of patients eventually relapse and confer resistance to platinum. Recent preclinical evidence on platinum-resistant ovarian cancer (PROC) is encouraging. Various potential mechanisms, such as genomic and epigenetic alterations, pharmacological alterations, DNA damage repair, metabolic reprogramming, the tumor microenvironment (TME) and programmed cell death, have been implicated in platinum resistance. In addition, clinical trials regarding the treatment of PROC have shown considerable success, and a multitude of promising therapies are in progress. In this review, we comprehensively summarized the underlying mechanisms of platinum resistance in OC and proposed the most promising novel therapeutics and strategies employed in the treatment of PROC.

## Introduction

Ovarian cancer (OC) constitutes one of the most fatal gynecologic malignancies in women. Globally, it is responsible for 3.4% of all malignancies and 4.7% of cancer-related deaths among females.^1^ In 2023, there would be approximately 19,710 new cases and 13,270 deaths from OC in the United States.^2^ Most OC patients suffer from “Silent Symptoms”, such as abdominal pain or swelling, causing them to miss the optimal treatment window.^3^ As a result, more than 70% of patients are diagnosed at an advanced stage and have a poor prognosis. Due to tumor heterogeneity, OC is subdivided into epithelial, germ cell, and sex cord-stromal subtypes. Epithelial ovarian cancer (EOC) accounts for approximately 90% of OC and includes serous (52%), endometrioid (10%), clear cell (6%), or mucinous carcinomas (6%), with a quarter being more rare or unspecified subtypes. Among them, high-grade serous ovarian cancer (HGSOC) is the most common.^4^^,^^5^

For newly diagnosed OC patients, the standard treatment is tumor cytoreductive surgery combined with chemotherapy. Due to their outstanding anti-tumor effects and relatively clear mechanisms of action, platinum analogues, especially cisplatin, carboplatin and oxaliplatin, are widely applied in the clinical treatment of OC.^6^ These drugs kill tumor cells by disrupting DNA replication and triggering cell apoptosis. Briefly, platinum-based drugs are transported into tumor cells via copper transporter 1 (CRT1).^7^ As the chloride ion concentration within tumor cells is much lower than that in the extracellular matrix, the chloride ligands of the platinum complexes are promptly substituted by water molecules or other small molecules containing thiol groups, resulting in the formation of active substances such as cis-[Pt(NH_3_)_2_Cl(OH_2_)]^+^ and cis-[Pt(NH_3_)_2_(OH_2_)_2_]^2+^. These active ingredients then bind preferentially to the N7 position of neighboring guanine (G) bases, generating intra- and inter-strand cross-links in DNA.^8^ The resulting structural distortion of DNA causes DNA damage and triggers a signaling cascade that leads to apoptosis.

The platinum-free interval (PFI) is defined as the period between the date of the last platinum dose administration and the date of disease relapse. It has been widely accepted and employed for predicting the response to platinum and for the selection and stratification of patient cohorts in trials. Based on PFI duration, the Gynecologic Cancer InterGroup (GCIG) consensus stated that patients could be categorized into four subgroups, namely, platinum-refractory (PFI ≤1 month), platinum-resistant (1 month < PFI ≤6 months), partially platinum-sensitive (6 months < PFI ≤12 months) and platinum-sensitive (PFI >12 months).^9^^,^^10^

Platinum resistance in OC can be divided into intrinsic platinum resistance and acquired platinum resistance. Intrinsic platinum resistance refers to tumors that fail to respond to initial platinum-based chemotherapy, leading to disease progression during first-line therapy or immediately after therapy. In contrast, acquired platinum resistance develops after an initial positive response to treatment.^11^ In fact, intrinsic platinum resistance usually occurs in certain subtypes of OC, including clear cell, transitional cell, mucinous, and LGSOC.^12^, ^13^, ^14^ These subtypes are infrequently treated with standard platinum-based chemotherapy; thus, there is relative paucity of research on the molecular mechanisms of chemotherapy resistance. However, approximately 80% of HGSOC patients initially respond well to platinum-based chemotherapy, so the first-line chemotherapy regimens for HGSOC patients are platinum analogues with the addition of a taxane (paclitaxel or docetaxel).^15^ Nevertheless, most of them will eventually relapse and progress to acquired platinum resistance.^16^

**Figure 1:**
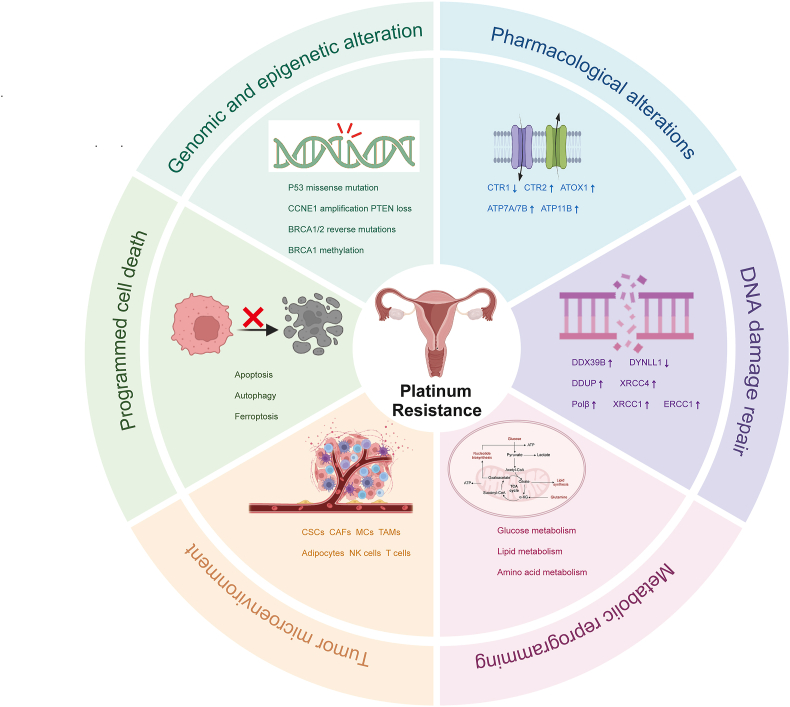
Schematic representation of the underlying mechanisms of platinum resistance in ovarian cancer.

Given that the majority of patients with PROC will suffer from poor clinical outcomes and that the response rate to second-line chemotherapy is lower than that of patients with platinum-sensitive ovarian cancer, it is crucial to identify the mechanisms of platinum resistance in OC. In this review, we summarized the underlying mechanisms of platinum resistance, including genomic and epigenetic alterations, pharmacological alterations, DNA damage repair, metabolic reprogramming, the tumor microenvironment (TME), and programmed cell death (Fig. 1). Additionally, we elaborated the possible treatment strategies to treat PROC patients and prolong their clinical outcome.

## Mechanisms of platinum resistance in OC

### Genetic and epigenetic alterations

The *TP53* gene plays a central role in apoptosis. In EOC, 96% of cases have *TP53* mutations,^17^ but the role of *TP53* mutations in platinum drug resistance remains controversial. By analyzing *TP53* mutations from 178 OC patients, Press et al found that patients with over-expression or missense mutations in *TP53* were more likely to experience platinum resistance than those with normal *TP53.*^18^ Meanwhile, patients with platinum-resistant EOC had more *TP53* mutations.^19^ However, a recent study suggested that *TP53* gain-of-function (GOF) mutations are not associated with platinum resistance in HGSOC.^20^ Hence, *TP53* mutations are complex, and each *TP53* mutation may have distinct consequences in different subtypes of OC, indicating that each mutation requires individual study.

*CCNE1* encodes the protein cyclin E1, which is responsible for the controlled transition from G1 to S phase. *CCNE1* amplification is prevalent among HGSOC patients with primary platinum resistance.^21^^,^^22^ Recent reports suggest that the WEE1 kinase inhibitor adavosertib is safe and has effective clinical activity in EOC with *CCNE1* amplification.^23^^,^^24^ Furthermore, it has been demonstrated in pre-clinical cancer models that PKMYT1 inhibition is a promising strategy for treating *CCNE1*-amplificated cancers.^25^

Phosphatase and tensin homolog deleted on chromosome 10 (*PTEN*) is known as a tumor suppressor by regulating the phosphatidylinositol 3-kinase (PI3K) pathway. *PTEN* is mainly down-regulated in human solid malignancies.^26^ A recent pathological study revealed that 21% of the patients have *PTEN* loss in HGSOC, which is correlated with worse PFS.^27^ Moreover, *PTEN* deficiency was found to be mutually exclusive with *CCNE1* amplification. The above study suggests that there might be a potential connection between *CCNE1* amplification and *PTEN* loss in HGSOC, but the underlying molecular mechanism remains to be further explored.

Accumulated epigenetic modifications, such as DNA methylation and modifications of histone marks, are also intimately linked to the development of platinum resistance.^28^^,^^29^ For example, promoter methylation of tumor suppressor genes (e.g., BRCA1, hMHL1, SLFN11, and COL1A) was implicated in acquired resistance to platinum.^22^^,^^29^, ^30^, ^31^ On the basis of this rationale, several clinical trials have been conducted and the results showed that hypomethylating agents (e.g., guadecitabine, decitabine, and azacitidine) can partially restore sensitivity to platinum agents in patients with PROC.^32^, ^33^, ^34^, ^35^, ^36^ Besides, transmembrane protein 88 (TMEM88), gelsolin and FANCF promoter hypomethylation are associated with platinum resistance in OC.^37^, ^38^, ^39^ Nuclear CD55 is reported to bind to and suppress ZMYND8 expression, thus derepressing PRC2 to increase H3K27me3, leading to platinum resistance in OC.^40^ C/EBPβ confers to platinum resistance by recruiting the methyltransferase DOT1L and reprogramming H3K79 methylation of multiple drug-resistance genes.^41^ In addition to methylation, histone modifications are also associated with PROC.^42^ Reduced global levels of H3K27me in cisplatin-resistant A2780/cp70 cells lead to resensitization to cisplatin.^43^ Recently, Sun et al demonstrated that histone lactylation, especially of H3K9la, was elevated in PROC and that inhibiting H3K9la enhanced the cisplatin response.^44^ They found that H3K9la enhances homologous recombination (HR) repair by directly activating RAD51 and BRCA1 expression, subsequently conferring cisplatin resistance.

### Pharmacological alterations

**Figure 2:**
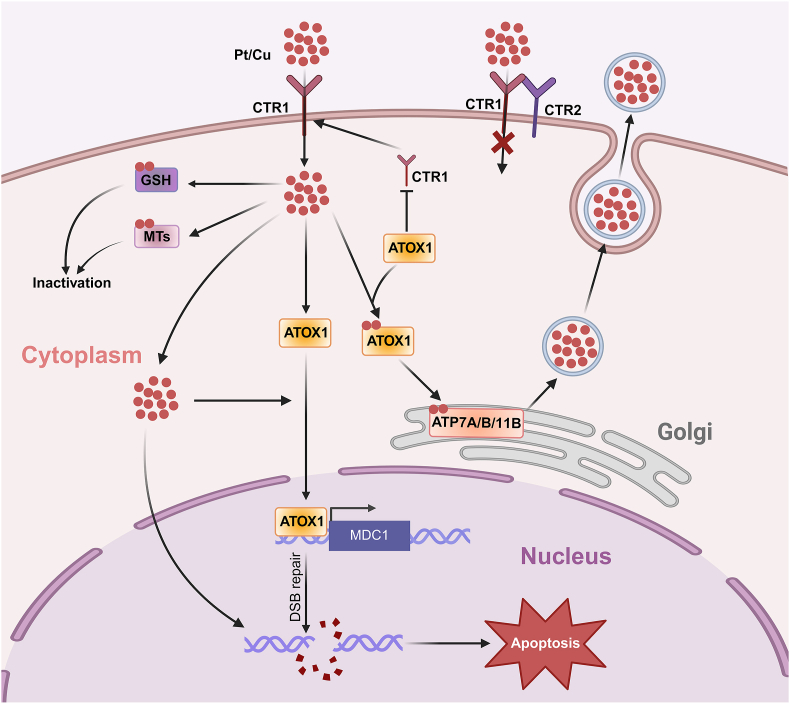
Schematic depiction of the association between the platinum distribution in tumor cells and platinum resistance in ovarian cancer. Low expression of CTR1 or high expression of CTR2 leads to impaired cisplatin uptake. Partial cisplatin taken up via CTR1 are transferred to ATOX1. Increased intracellular cisplatin induces the expression of ATOX1. ATOX1 can induce the polyubiquitination and down-regulation of CTR1, leading to reduced cisplatin uptake. Besides, ATOX1 transfers cisplatin to ATP7A/B and ATP11B in the secretory pathway, which leads to the sequestration and extrusion of cisplatin in vesicular compartments. Intriguingly, exposure to platinum agents promotes the entry of ATOX1 into the nucleus to increase the transcription of MDC1, thereby promoting DNA damage repair. Moreover, GSH and MTs can bind to cisplatin, leading to its inactivation.

In general, reduced intracellular accumulation due to impaired uptake and/or augmented efflux is considered one of the causes of platinum resistance (Fig. 2). Some evidence imply that the influx and efflux transporters involved in maintaining copper homeostasis also participate in the transportation of platinum agents.^45^ It has been convincingly demonstrated that CTR1 (also known as SLC31A1), the major copper influx transporter, controls the uptake of platinum agents. Loss of CTR1 confers platinum resistance by reducing the uptake of platinum agents.^46^^,^^47^ Conversely, low levels of CRT2 have been suggested to be associated with increased platinum accumulation throughout the cell and higher sensitivity to platinum.^47^^,^^48^ CRT2 functions primarily in regulating the biogenesis of an inactive form of CTR1 that lacks the copper- and cisplatin-binding ectodomain. Thus, it regulates platinum accumulation and sensitivity in a CRT1-dependent manner.^48^

In addition to CRT1 and CRT2, ATP7A and ATP7B are involved in the efflux of platinum agents.^49^ Accumulating evidence suggests that ATP7A and ATP7B contribute to the emergence of cisplatin resistance in OC. Furthermore, the overexpression of ATP7A and ATP7B is strongly associated with unfavorable clinical outcomes and poor chemotherapy response in OC.^50^, ^51^, ^52^ They can interact directly with cisplatin to trigger its relocalization and facilitate the sequestration of cisplatin in the vesicular structures of the secretory pathway, therapy preventing its binding to genomic DNA.^53^, ^54^, ^55^, ^56^ Like ATP7A and ATP7B, ATP11B was recently demonstrated to show the same properties in OC. It colocalizes with cisplatin and contributes to the efflux of cisplatin via the secretory vesicular pathway.^57^

Antioxidant 1 copper chaperone (ATOX1) is a key copper that delivers copper to the metal-binding domains (MBDs) of the ATP7A and ATP7B proteins. It is generally believed that the active component formed by cisplatin entering cancer cells can bind to ATOX1 and that their interaction rates are limited by dichlorination.^58^^,^^59^ Extensive studies have shown that ATOX1 is a candidate for cisplatin resistance,^59^^,^^60^ and its high expression mainly results in cisplatin resistance through two approaches. On the one hand, cisplatin exposure induces the polyubiquitination and down-regulation of CTR1 in an ATOX1-dependent manner, leading to reduced cisplatin uptake. On the other hand, ATOX1 transfers cisplatin to ATP7A and ATP7B in the secretory pathway, which leads to the sequestration and extrusion of cisplatin in vesicular compartments and blocks the binding of cisplatin to DNA.^60^ Intriguingly, another possible mechanism of ATOX1-mediated cisplatin resistance was recently discovered. It has been suggested that exposure to platinum agents promotes the entry of ATOX1 into the nucleus to increase the transcription of MDC1, thereby promoting double-stranded DNA damage repair in a copper-dependent manner.^61^

Furthermore, intracellular platinum agents can conjugate to thiol-containing substances, such as the cytoplasmic tripeptide glutathione (GSH) and metallothioneins (MTs), leading to their inactivation prior to DNA binding.^62^ It has been reported that platinum agents bind to GSH and compete with ATOX1, leading to the intracellular inactivation of platinum agents.^63^ Additionally, there are indications that high intracellular levels of GSH and MTs are associated with platinum resistance and that a reduction in the levels of GSH and MT levels sensitizes cisplatin-resistant OC cell lines.^64^, ^65^, ^66^

### DNA damage repair

DNA damage response (DDR) is of paramount importance in maintaining genomic stability and cell survival.^67^ When tumor cells suffer from DNA damage, DDR is initiated, and specific DNA repair pathways, such as HR repair, non-homologous end joining (NHEJ) repair, base excision repair (BER), nucleotide excision repair (NER), mismatch repair (MMR), translesion DNA synthesis (TLS), and interstrand crosslink (ICL) repair are triggered to mend the damage.^68^ It is widely acknowledged that DNA is the principal target of platinum drugs, and efforts to eliminate platinum adducts and repair DNA damage play a fundamental role in the cytotoxicity of platinum drugs.^69^ Currently, a growing body of evidence has demonstrated that the restoration of DNA damage repair pathways is closely associated with platinum resistance in OC.^70^, ^71^, ^72^, ^73^

During the S and G2 phases of the cell cycle, HR functions as the predominant process to repair DNA double-strand breaks (DSBs).^74^ HR-related genes, particularly *BRCA1* and *BRCA2*, are crucial for guaranteeing the error-free repair of DSBs.^75^ However, germline mutations in *BRCA1/2* (gBRCAm) and somatic mutations of *BRCA1/2* (sBRCAm) predispose to OC.^76^ Additionally, homologous recombination repair deficiency (HRD) occurs in approximately 50% of HGSOC patients,^21^^,^^77^ which could be one of the reasons for the favorable initial response of most HGSOC patients to platinum-based chemotherapy. Secondary somatic mutations restoring *BRCA1/2* are significantly associated with chemotherapy resistance.^78^, ^79^, ^80^, ^81^, ^82^ Furthermore, whole-genome analyses of PROC indicated that multiple independent reversions of the *BRCA1/2* mutations as well as the loss of *BRCA1* promoter methylation both exert significant influences on the acquisition of platinum resistance.^22^ Recently, several molecules regulating HR signaling have also been reported to be associated with platinum resistance. DDX39B binds to and stabilizes *BRCA1* mRNA, ensuring the accumulation of RAD51 at DSB sites and facilitating the HR pathway. It has been revealed that high expression of DDX39B leads to hyposensitivity to platinum agents and is correlated with worse survival in OC patients.^83^ DYNLL1 is identified to bind directly to MRE11 to reduce its end-resection activity. Loss of DYNLL1 restores the HR signaling pathway and thereby induces platinum resistance.^84^ Moreover, the DDUP microprotein encoded by the CTBP1-DT lncRNA increases drastically in PROC and is inversely correlated with the response to cisplatin-based therapy. In particular, cisplatin-induced DDUP foci sustains the RAD18/RAD51C complexes and promotes the HR pathway.^85^ Recently, it was discovered that YBX1 is highly expressed in PROC-derived organoids, and YBX1 can promote HR and platinum resistance by recognizing m5C modification in CDH3 and enhancing its mRNA stability.^86^ LINC02776 has also been identified as a critical factor of PROC. Mechanistically, high HIF-1α in platinum-resistant tissues stimulates the expression of LINC02776, which confers to platinum resistance by directly binding to PARP1, promoting PARP1-dependent polyADP-ribosylation and restoring HR repair.^87^ These findings demonstrate that the HR pathway plays an important role in platinum resistance.

In addition to HR restoration, other DNA repair pathways also participate in platinum resistance. Cisplatin promotes the translocation of the JNK-cJUN complex into the nucleus, leading to the activation of XRCC4 (key gene of NHEJ) and XRCC1 (key gene of NER) transcription to confer cisplatin resistance.^88^^,^^89^ DNA polymerase β (Polβ), a crucial player in BER, is highly expressed in PROC cell lines, and its depletion can increase sensitivity to platinum.^89^ Furthermore, the NER signaling pathway contributes to repairing DNA bound to cisplatin, and disrupting the NER signaling pathway can substantially enhance cisplatin cytotoxicity *in vivo* and *in vitro.*^90^ Both the expression of ERCC1 and the genotype of the ERCC1 polymorphism Asn118Asn could serve as biomarkers for predicting platinum resistance in EOC patients.^91^ However, recent studies suggest that the knockdown of NER factors has only a minimal effect on cisplatin sensitivity.^92^ Moreover, MMR deficiency in hMutL alpha and hMutS alpha confers cisplatin resistance by preventing futile cycles of TLS and mismatch correction.^93^ Meanwhile, it has been reported that hMSH2 hypermethylation and its low expression are strongly associated with platinum resistance in EOC.^94^ OTUB1 inhibits the ubiquitination of hMSH2 by blocking E2 ligase ubiquitin transfer activity. The degradation of hMSH2 caused by depletion of OTUB1 also confers cisplatin resistance.^95^

Recently, the role of TLS and ICL repair in platinum resistance has drawn escalating attention. It has been demonstrated that the intrinsically enhanced Pol η-mediated TLS in ovarian cancer stem cells (CSCs) might be one of the causes for the chemoresistance traits exhibited by CSCs.^96^ Moreover, the function of tyrosine-derived fumarate in modulating TLS and chemosensitivity in EOC has been uncovered. Fumarylacetoacetate hydrolase (FAH) is an enzyme that catalyzes the final step of tyrosine catabolism and its expression significantly correlated with chemotherapy efficacy in patients with EOC. FAH-produced fumarate suppressed TLS by binding directly to REV1, resulting in the improvement of chemosensitivity. Surprisingly, *in vivo* tyrosine supplementation reduced the incidence of chemotherapy resistance and improved its sensitivity.^97^ Compared to OC patients after platinum-based chemotherapy, patients before platinum-based chemotherapy show a lower proportion of repair (unhooking) of DNA interstrand crosslinks, indicating that platinum-based chemotherapy induces increased ICL repair and clinical acquired resistance in OC.^98^ Additionally, AND-1 phosphorylation at T826 is increased in cisplatin-resistant OC cells, and its high phosphorylation leads to cisplatin by promoting ICL repair in OC. Mechanistically, AND-1 is phosphorylated at T826 in a manner dependent on Ataxia Telangiectasia Mutated (ATM) and Rad3-related proteins. Subsequently, the phosphorylated AND-1 rapidly accumulates at the ICL-stalled fork and interacts with FANCM/FAAP24, resulting in the recruitment of the FANCM/FAAP24 complex to ICLs and the promotion of ICL repair.^99^

### Metabolic reprogramming

Metabolic reprogramming is a hallmark of cancer cells. It can not only help cancer cells adapt to the increased energy demands for limitless proliferation but also endow themselves with a chemoresistance phenotype.^100^ Metabolic alterations, including glucose metabolism, lipid metabolism, and amino acid metabolism, have been associated with platinum resistance in OC.

It is well known that cancer cells prefer to utilize glucose through aerobic glycolysis.^100^ Most regulators of glycolytic activity, including glucose transporter 1 (GLUT1), hexokinase 2 (HK2), M2 isozyme of pyruvate kinase (PKM2), and lactate dehydrogenase A (LDHA), were highly expressed and associated with a poor prognosis in OC.^101^^,^^102^ Mounting literatures report that aerobic glycolysis is one of the causes for platinum resistance.^103^^,^^104^ HK2 confers resistance to cisplatin, and its depletion markedly improved cisplatin sensitivity by facilitating a shift towards oxidative glucose metabolism.^105^ In addition to glycolytic enzymes, several other genes confer platinum resistance by regulating glycolysis. It was suggested that Anexelekto (AXL) is highly expressed in the cisplatin-resistant OC cells A2780/DDP compared to the parental A2780 cells. It phosphorylates PKM2 at the Y105 site, leading to its increased activity. This interaction reduces the binding of PKM2 to PEP, thereby promoting glycolysis and leading to cisplatin resistance.^106^ Aurora-A, a member of the Aurora kinase family, regulates glycolysis to induce cisplatin resistance by promoting the SOX8/FOXK1 signaling axis by directly binding to the transcription factor SOX8 and phosphorylating the Ser327 site.^107^ Wilms' tumor 1-associating protein (WTAP) is reported to contribute to PROC through regulating the conversion from aerobic glycolysis to oxidative phosphorylation by promoting the expression of ME1, which is a rate-limiting enzyme that regulates the shuttle of malic acid in mitochondria and cytoplasm.^108^ Recent studies have suggested that lactate derived from aerobic glycolysis contributes a lot to chemotherapy resistance.^109^, ^110^, ^111^ In ovarian cancer, chemotherapy impairs glucose uptake and decreases the intracellular glucose level, which triggers the acetylation of ME2 at lysine 156 by ACAT1, leading to increased ME2 enzyme activity and facilitating the production of lactate from glutamine.^112^ Excess lactate promotes the activity of HR repair-related genes and drives the ESM1-CD1 axis to induce cisplatin resistance.^112^^,^^113^

Recently, more and more attention has been paid to the effect of lipid metabolism on chemoresistance.^114^^,^^115^ Researchers have revealed that fatty acid oxidation (FAO) is intimately correlated with platinum resistance. High-throughput stimulated Raman scattering imaging and single cell analysis found that fatty acid uptake by cisplatin-resistant cells is augmented and that the increased fatty acid uptake enhances beta-oxidation, thereby ensuring cancer cells survival under cisplatin-induced oxidative stress.^116^ Meanwhile, via multi-omics analysis, higher expression of FAO pathway-related genes was identified in cisplatin-resistant HGSOC cells. Both *in vivo* and *in vitro*, by suppressing CPT1A (a key rate-limiting step of FAO) or employing CRISPR knockout technology, the sensitivity of HGSOC cells to platinum drugs can be enhanced.^117^ Besides, COL11A1 results in cisplatin resistance, which is accomplished by increasing fatty acid synthesis and FAO.^118^ Heterozygous deletion of NKX2-8 is prevalent, and loss of NKX2-8 also confers platinum resistance in EOC. Mechanistically, loss of NKX2-8 led to reprogramming of fatty acid metabolism in the adipose microenvironment through epigenetic regulation of the expression of CPT1A and CTP2.^119^ Furthermore, cholesterol metabolism dysregulation is responsible for platinum resistance in OC. It has been reported that platinum-resistant OC cells rely primarily on the uptake of exogenous cholesterol to meet their needs, while endogenous cholesterol biosynthesis is reduced.^116^ In OC cells with platinum resistance, the expression of FDPS and OSC enzymes, which are related to cholesterol synthesis, is decreased, whereas the expression of LDLR, which is related to cholesterol uptake, is increased.^120^ Recently, it has been proved that platinum-resistant OC cells increase cholesterol uptake by increasing SR-B1. SR-B1 blockade diminishes cholesterol uptake and accumulation in cells, and triggers lipid oxidative stress by reducing the expression of GPX4, leading to ferroptosis. GPX4 depletion, in turn, reduces cholesterol uptake by SR-B1, and resensitizes platinum-resistant OC cells to platinum through the transcriptional regulation of SREBF2. SREBF2 participated in the process of cholesterol metabolism, and the inhibition of SREBF2 resulted in decreased SR-B1 expression and cholesterol uptake.^121^ These could be the possible mechanisms by which how cholesterol regulates platinum sensitivity.

In addition, the reprogramming of amino acids, including serine,^122^ glutamine,^123^ and glutathione (GSH),^124^ contributes to tumor adaptation to environmental insults and platinum resistance. It was uncovered that PROC cells existed decreased serine biosynthetic activity and PHGDH expression, which mediated the biosynthesis of serine from glucose. The down-regulation of PHGDH could aid in sustaining PARP activity under platinum exposure.^122^ All the evidence indicates that serine metabolism may serve as an actionable vulnerability in OC adaptation after platinum exposure.^122^ Moreover, PROC cells are enriched in intracellular glutamine and GSH, and the deprivation of glutamine and GSH promotes the chemotherapy response through ROS accumulation, detoxification reduction, and ferroptosis.^65^^,^^123^^,^^125^^,^^126^

### Tumor microenvironment

**Figure 3:**
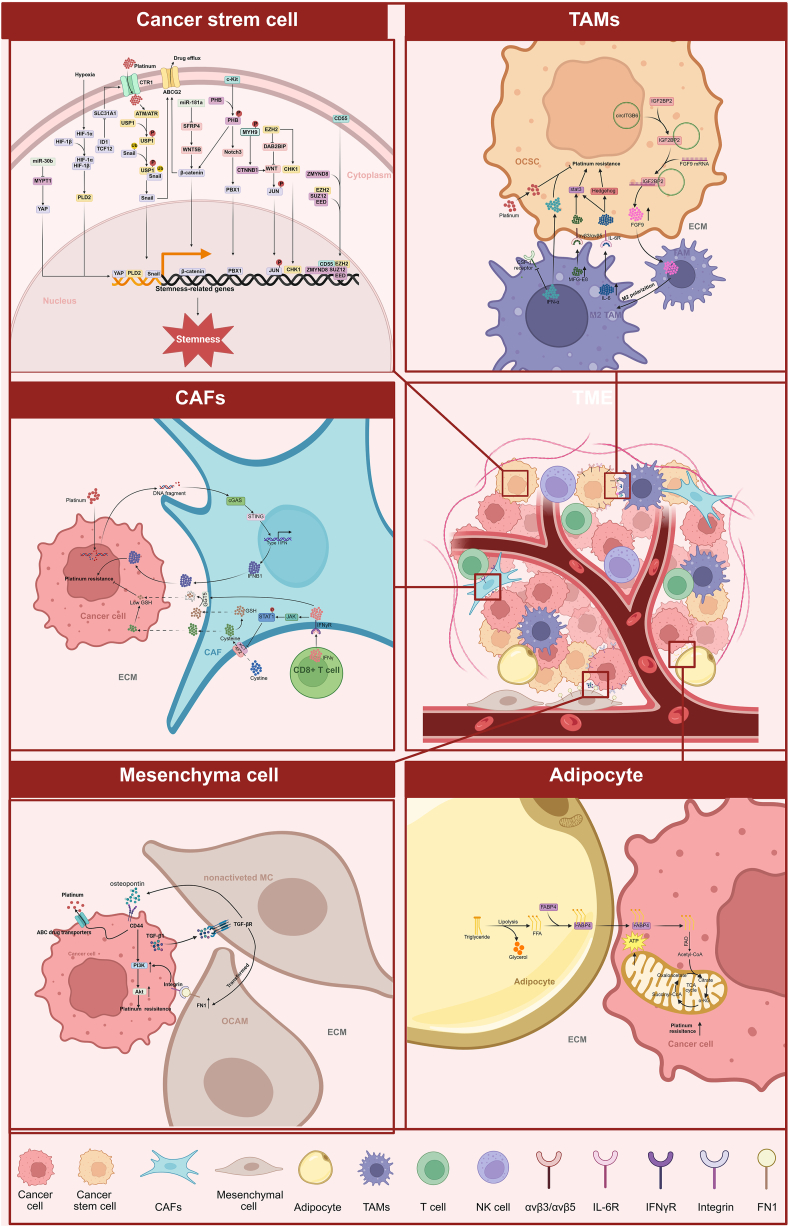
Influence of the TME on ovarian cancer platinum resistance. Ovarian cancer is enriched with CSCs, and stem cell subpopulations contribute to tumor chemoresistance via different pathways, including impaired uptake and increased efflux. The circRNA circITGB6 induces the polarization of TAMs toward the M2 phenotype through the formation of a ternary circITGB6/IGF2BP2/FGF9 RNA‒protein complex in the cytoplasm, resulting in cisplatin resistance. Polarized TAMs produce large amounts of MFG-E8 and IL-6, which synergistically mediate cisplatin resistance through the coordinated activation of stat3 and sonic hedgehog signaling. Platinum-induced genomic instability drives DNA transfer from cancer cells to cancer-associated fibroblasts (CAFs), leading to the activation of the cGAS-STING-IFNB1 signaling pathway in CAFs. Consequently, increased IFNB1, glutathione, and cysteine release in CAFs reinforce platinum resistance of OC cells. However, CD8^+^ T cell could overcome the resistance by altering glutathione and cystine metabolism in fibroblasts via the IFNγ/JAK/STAT1 pathway. Mesothelial cells (MCs) confer platinum resistance by promoting the PI3K/Akt signaling pathway via cell–cell interactions. Adipocytes mediate carboplatin resistance by inducing the FABP4-mediated FAO pathway.

In addition to malignant cells, the cellular compartment of the TME is composed of mesenchymal cells, adipocytes, immune cells and ovarian cancer stem cells (OCSCs). Besides, the TME of OC patients is heterogeneous among patients with various tumors in one patient and even within distinct locations of a single tumor (Fig. 3).^127^ A growing number of studies suggest that the interactions between tumor cells and stromal cells within the TME affect disease development and the response to therapy.^128^^,^^129^ Using the transcriptional profiles of advanced EOC samples, Hao et al established an immune score that consists of marker genes related to specific immune cell subtypes. Their results showed that a higher immune score reflected higher expression of favorable prognostic genes, and patients with higher immune scores responded better to chemotherapy.^130^ More importantly, PROC is clearly characterized by reduced infiltration of CD8^+^ T cells and activated CD4^+^ T cells.^131^ Single-cell RNA sequencing analysis indicated that the PROC group exhibited a higher proportion of epithelial cells, cancer-associated fibroblasts (CAFs), macrophages/monocytes, and endothelial cells, and a lower proportion of CD4^+^ T cell, CD8^+^ T cells and B cells.^132^, ^133^, ^134^ In particular, FBXO2^+^ epithelial cells and TACSTD2^+^ epithelial cells were identified to contribute to PROC.^134^^,^^135^ Bedsides, B7–H4, an immune checkpoint protein, contributed to immune evasion and showed a strong association with PROC.^136^ However, data on the TME of PROC are scarce at present. There is an urgent need for a comprehensive characterization of the TME landscape of PROC.

It is known that the persistence of OCSC is responsible for platinum resistance in OC.^137^ Previous evidence has demonstrated that EZH2 contributes to chemoresistance by maintaining the properties of OCSC. EZH2 regulates OCSC stemness mainly through two pathways, one by activating the CHK1 signaling pathway and the other by regulating the DAB2BIP/Wnt/c-JUN signaling pathway.^138^^,^^139^ It has been revealed that HOXC transcript antisense RNA (HOTAIR) recruits enhancer of EZH2 to catalyze H3K27m3 to promote the phenotype of OCSC, subsequently leading to PROC. Combining a HOTAIR inhibitor and an EZH2 inhibitor is a promising therapeutic strategy to resensitize ovarian cancer cells to platinum-based chemotherapy.^140^ In addition, it has been reported that the miR-181a/SFRP4 axis and SPC25/RIOK1/MYH9 axis promote stemness and platinum resistance by activating the Wnt signaling pathway in OC.^141^^,^^142^ c-Kit binds directly to PHB and facilitates its phosphorylation at tyrosine (phospho-PHB^Y259^) in the membrane raft. The raft-phospho-PHB^Y259^ enhances OC stemness and chemoresistance via the Notch3/PBX1 and β-catenin/ABCG2 signaling pathways.^143^ Snail transcription factors have been implicated in OC stemness and chemoresistance.^144^ Recently, USP1 was reported to confer platinum resistance by enhancing the stability of the snail protein. After platinum treatment, USP1 is phosphorylated by ATM and ATR, and phosphorylated USP1 binds to the snail protein. Consequently, USP1 deubiquitinates and stabilizes the snail protein expression, leading to increased stem cell-like features and acquired resistance to platinum.^145^ MiR-30b is overexpressed in cisplatin-resistant OC cells, and the miR-30b/MYPT1 axis regulates cell stemness and chemotherapy sensitivity through the activation of the Hippo signaling pathway.^146^ Furthermore, CD55 is found to localize the nucleus of ascites from PROC patients and is enriched in chemoresistant OC cells. In the nucleus, CD55 binds to chromatin and interacts with ZMYND8 and the PRC2 complex, leading to the induction of cancer cell stemness and chemoresistance.^40^ Hypoxia in solid tumors is an important source of chemoresistance. It has been suggested that hypoxia could induce chemoresistance by promoting OCSC formation through transcriptional activation of the PLD2 gene.^147^ Recently, it was reported that OCSCs exhibit increased basal autophagy compared to non-OCSCs. In OCSCs, high levels of ID1 reduce CTR1 expression and cisplatin influx by sequestering TCF12, thereby leading to cisplatin resistance.

Mesothelial cells (MCs) serve as the initial barrier meeting metastatic OC cells.^148^ It has been suggested that MCs confer platinum resistance by promoting the FN1/Akt signaling pathway via cell–cell interactions.^149^ In addition, the MC-derived key secreted factor osteopontin can facilitate platinum resistance via the activation of the CD44 receptor, PI3K/AKT signaling, and ABC drug efflux transporter activity.^150^ It's worth mentioning that PROC grows as a metastatic disease, disseminating in the abdomen and pelvis. Compared to platinum-sensitive ovarian cancer cells, platinum-resistant ovarian cancer cells commonly have a greater ability to adhere to and grow on MCs in layer.^151^ With the stimulation of platinum, overexpressed ITGA6 in platinum-resistant ovarian cancer cells is secreted into the extracellular space and activates the IGF1R-Src pathway, resulting in the promotion of cancer cell dissemination.^152^ Based on the above research results, the inhibition of MC/OC adhesion might be a promising therapeutic intervention to prevent PROC dissemination and improve the platinum response.^152^^,^^153^ Recently, cancer-associated fibroblasts (CAFs) were reported to play a pivotal role in chemotherapy resistance in OC. CAFs promote cisplatin resistance in an indirect non-contact manner. Mechanistically, platinum-induced genomic instability drives DNA transfer from cancer cells to CAFs, then activating the cGAS-STING-IFNB1 signaling pathway in CAFs. Consequently, increased IFNB1 release reinforces platinum resistance of OC cells.^154^ In addition, glutathione and cysteine released by CAFs contribute to this resistance. However, CD8^+^ T cells could overcome the resistance by altering glutathione and cystine metabolism in fibroblasts via the IFNγ/JAK/STAT1 pathway.^155^ Lengyel and his colleagues have revealed that adipocytes not only promote ovarian cancer metastasis but also mediate carboplatin resistance by inducing the expression of the lipid chaperone protein FABP4 and thereby regulating the FAO pathway to confer carboplatin resistance.^156^

Several studies have indicated that tumor-associated macrophages (TAMs) are associated with platinum sensitivity. TAMs can reversibly form two main phenotypes: the M1-like and M2-like phenotypes. Previous studies have demonstrated that high levels of M2-like TAMs and low M1/M2 ratios infiltrating tumors predict poor clinical response to chemotherapy in OC.^157^^,^^158^ CircRNA circITGB6 induced TAMs to polarize to the M2 phenotype through the formation of a ternary circITGB6/IGF2BP2/FGF9 RNA–protein complex in the cytoplasm, resulting in cisplatin resistance of OC.^159^ TAMs produce large amounts of MFG-E8 and IL-6, which synergistically mediate cisplatin resistance through the coordinated activation of STAT3 and sonic hedgehog signaling in OCSC populations.^160^ Besides, therapeutic targeting of macrophages by blocking CSF-1R signaling improves the efficacy of cisplatin therapy by unleashing type Ⅰ interferon response.^161^ In addition to preclinical studies, Yeku OO et al constructed a phase 1 clinical trial to investigate the effects of TAMs and myeloid-derived suppressor cells (MDSCs) in PROC. Patients with PROC were treated with PY159, a monoclonal antibody that binds to TREM1 expressed on TAMs and MDSCs, leading to repolarization towards an inflammatory and anti-tumor phenotype, and PY314, a monoclonal antibody that binds to TREM2 on TAMs, leading to TAM depletion, as a single agent and in combination with pembrolizumab. Both PY159 and PY314 led to a 50 % clinical benefit rate in PROC.^162^ All the evidence suggested that targeting TAMs might be a promising therapeutic strategy to treat PROC, and more clinical trials are needed. Moreover, NK cells and Treg cells might also play important roles in platinum resistance of OC.^163^ However, the potential mechanisms are still unrevealed. RNA sequencing of tumor small extracellular vesicles (sEVs) revealed that the lncRNA CATED is overexpressed in tumor-derived sEVs in PROC and that its high expression is significantly associated with a poor prognosis. Functionally, CATED conferred to platinum resistance by inhibiting apoptosis via the DHX36-RAP1-MAPK pathway.^164^

### Programmed cell death

Platinum binds to DNA to produce intra- and inter-strand crosslinks and induces DNA damage culminating in mitochondria-mediated apoptosis. Therefore, the effectiveness of platinum-based chemotherapy depends heavily on the ability of cancer cells to undergo drug-induced apoptosis. Apparently, failure to engage in apoptosis appears to be one of the main mechanisms of resistance to platinum. Inhibition of apoptosis-related proteins increase cisplatin resistance in OC cells.^165^, ^166^, ^167^, ^168^ Ferroptosis has been indicated to be associated with platinum resistance, and targeting ferroptosis holds great potential to overcome chemotherapy resistance in OC.^169^^,^^170^ Both SLC7A11 and GPX4 are central regulators of ferroptosis. Cheng et al discovered that both SCL7A11 and GPX4 were up-regulated in PROC cells compared to their parental OC cells. Moreover, patients with high co-expression levels of SLC7A11 and GPX4 had a 60-fold higher risk of platinum resistance and a worse prognosis.^171^ Wang et al found that FZD7 marks PROC cancer cells with stemness and ferroptosis characteristics. The overexpression of FZD7 activated TP63, leading to the transcriptional up-regulation of GPX4, thus protecting cells from the oxidative stress induced by platinum.^172^ SCD1 and FADS2, two critical fatty acid desaturases, are also responsible for cisplatin resistance by regulating ferroptosis. Combination treatment with SCD1/FADS2 inhibitors can synergistically enhance cisplatin sensitivity.^173^ In addition, ACSL1 has been shown to contribute to platinum resistance. Platinum chemotherapy increases the expression of ACSL1, which can myristoylate the FSP1 protein and inhibit its degradation. The increased FSP1 protein counteracts ferroptosis and induces platinum resistance.^174^ Autophagy can prevent tumorigenesis and induce chemotherapy resistance as an adaptive response.^175^ Previous studies have demonstrated that platinum drugs trigger autophagy, which is one of the mechanisms of chemoresistance.^176^^,^^177^ Multiple genes have been proved to confer platinum resistance by inducing autophagy, and pharmacological inhibition of autophagy may serve as a potential therapeutic strategy to combat platinum resistance in OC.^178^, ^179^, ^180^, ^181^, ^182^, ^183^, ^184^, ^185^ Dysregulation of pyroptosis, an inflammation-linked programmed cell death mediated by gasdermins, has also been shown to affect platinum resistance. It was recently reported that cytokine receptor-like factor 1 (CRLF1) enhances chemoresistance by inhibiting AKT/SIN1-dependent pyroptosis.^186^

### Treatment approaches for PROC

The mainstay of treatment for PROC patients encompasses the sequential application of cytotoxic agents, including pegylated liposomal doxorubicin (PLD), gemcitabine, paclitaxel, and topotecan. It has been disclosed that the objective response rates (ORRs) of these medications range from 10 % to 15 %, with an approximate median progression-free survival (PFS) of 3.5 months and a median overall survival (OS) of approximately 12 months.^187^ The therapy choice essentially depends on the previous treatments, treatment costs, patients’ physical state, and feasibility. In particular, once-weekly administration appears to be an effective treatment strategy with better tolerability and fewer adverse drug effects. Bevacizumab, a recombinant humanized monoclonal antibody targeting VEGFA, represents another option for treating PROC patients. In the AURELIA open-label randomized phase Ⅲ trial, the addition of bevacizumab to chemotherapy significantly improved PFS and ORR. In 2014, bevacizumab has been proved to treat PROC in association with chemotherapy.^188^

### Antibody–drug conjugates (ADCs)

Recently, antibody–drug conjugates (ADCs) have gained escalating prominence within the area of oncology drug development, particularly in the context of treating PROC. Mirvetuximab soravtansine (MIRV), a first-in-class antibody–drug conjugate targeting the folate receptor α (FRα), was granted accelerated approval by the U.S. Food and Drug Administration (FDA) in November 2022 for the treatment of patients with FRα positive, platinum-resistant epithelial ovarian, fallopian tube, or primary peritoneal cancer who received 1–3 prior systemic treatment regimens. In the SORAYA phase II study, 106 FRα-high expressing PROC patients who had received up to three prior therapies were enrolled. The results suggested that MIRV has clinically meaningful antitumor activity, with an ORR of 32.4% (95% CI, 23.6–42.2) and a median duration of response of 6.9 months (95% CI, 22.4–49.9).^189^ In the confirmatory MIRASOL phase Ⅲ study, 453 high FRα expressing PROC patients with 1–3 priors were randomized into the MIRV group and the investigator's choice of chemotherapy (IC) group. MIRASOL reported an mPFS of 5.62 months with MIRV versus 3.98 months with IC (HR, 0.65 [95% CI, 0.52–0.81; *p* < 0.0001]) and an ORR of 42.3% with MIRV versus 15.9% with IC (*p* < 0.0001). Excitingly, MIRV was the first reported drug to improve OS in a PROC trial, with an mOS of 16.46 months (HR, 0.67 [95% CI, 0.50–0.88; *p* = 0.0046]).^190^ Although the ORR of MIRV was relatively high in high FRα expressing PROC patients, it was still lower than certain expectations. The possible reasons for this phenomenon include tumor heterogeneity, MIRV resistance, the tumor microenvironment, and individual differences (metabolic capacity, immune status, etc.). In addition, PROC patients receiving MIRV treatment may experience some common adverse reactions, including eye discomfort (such as blurred vision and dry eyes), nausea, vomiting, fatigue, and rash. Among these, ocular toxicity is the most common adverse reaction, with an incidence rate of over 30%. Other common adverse reactions include gastrointestinal reactions (such as nausea and diarrhea) and hematological adverse reactions (such as anemia and leukopenia), with an incidence rate typically between 10% and 20%. In addition to these common adverse reactions, MIRV may also cause some serious adverse reactions, such as severe allergic reactions and liver function abnormalities. Although these occurrences are relatively rare, close monitoring is still required.

At present, a phase Ⅲ clinical trial (NCT05870748) assessing the efficacy of the FRα-directed ADC, luveltamab tazevibulin, in the treatment of PROC is recruiting patients. According to the National Comprehensive Cancer Network (NCCN) guidelines for the treatment of ovarian cancer (version 2024), trastuzumab deruxtecan (T-DXd), a human epidermal growth factor 2 (HER2)-directed ADC, is approved to treat PROC patients with HER2 IHC 3+/2+ expression. In the DESTINY-PanTumor02 phase II trial, T-DXd showed durable clinical benefits and meaningful survival outcomes. Specifically, in OC patients with confirmed HER2 IHC 3+ expression, the investigator-assessed ORR was 63.6% (95% CI, 30.8–89.1), and the mPFS was 12.5 months (95% CI, 3.1‒not reached).^191^ However, MIRV is only applicable to 35% of tumors with high FRα expression, and the prevalence of HER2 positivity in EOC is approximately 20%.^192^^,^^193^ Hence, researchers are trying to figure out other tumor-associated antigens.

Since CDH6 is over-expressed in approximately 65%–85% of OC patients and is associated with poor prognosis,^194^ the emerging CDH6-directed ADC raludotatug deruxtecan (R-DXd; DS-6000) was developed. According to the initial subgroup analysis results of a phase Ⅰ trial (NCT04707248), it provided a safe and effective clinical response in patients with heavily pretreated PROC. In this clinical trial, 50 patients were treated with R-DXd at doses ranging from 4.8 mg/kg to 8.0 mg/kg. Preliminary efficacy data presented an ORR of 46% (95% CI, 32%–61%). An additional efficacy assessment suggested that the disease control rate (DCR) with R-DXd was 98%. With a median follow-up of 5.8 months, the median duration of response was 11.2 months (95% CI, 3.0‒not reached). The mPFS was 7.9 months (95% CI, 4.4–12.4) at a median follow-up of 5.6 months.^195^ A phase 2/3 study of R-DXd in PROC patients (REJOICE-Ovarian01) is currently underway.^196^

### Immunotherapy

Due to the contribution of the TME to platinum resistance, immunotherapy is supposed to be effectively used to treat PROC. Nevertheless, it has been reported that the TME within EOC is profoundly immunosuppressive.^197^ Additionally, most trials treating PROC with immune checkpoint blockers (ICBs) with or without chemotherapy (e.g., NINJA, JAVELIN 200 Ovarian), have shown no effective clinical activity.^198^^,^^199^ However, several trials are still in progress in PROC. Particularly, the combination of nemvaleukin alpha and pembrolizumab has exhibited promising clinical activity in PROC, suggesting that immunotherapy may be a feasible approach in PROC. Nemvaleukin alpha plus pembrolizumab induced a remarkable expansion of CD8^+^ T cells and NK cells. The ORR was 28.6%, and the DCR was 71.4%.^200^ In 2021, the FDA granted fast track designation to nemvaleukin alfa plus pembrolizumab in PROC patients based on findings from the ARTISTRY-7 phase Ⅲ trial (NCT05092360). Currently, a phase Ib study of anlotinib plus TQB2450 (PD-L1 inhibitor) for PROC or platinum-refractory ovarian cancer has yielded exciting results. In this study, the ORR was 47.1% (95% CI, 29.8–64.9), and the DCR is 97.1% (95% CI, 84.7–99.9).^201^ A phase Ⅲ randomized controlled trial is currently in progress (NCT05145218). In addition, the safety and preliminary value of PY159 (an agonist antibody to TREM1) and PY314 (an agonist antibody to TREM2) were evaluated in RPOC patients. The results showed that both PY159 and PY314 were well tolerated and led to stable disease. The mPFS was 2.76 months (95% CI, 1.35–5.32) and 2.69 months (95% CI, 1.18–4.07) in PY159 and PY314, respectively.^162^ Recently, the EORTC 1508-GCG phase II study showed that bevacizumab plus atezolizumab numerically improved PFS in PROC. The mPFS and response rate were 4.1 months and 19%, respectively, for bevacizumab plus atezolizumab.^202^

### Ongoing phase Ⅲ clinical trials

In addition to the above studies, there are multiple ongoing phase Ⅲ clinical trials to improve therapeutic options for patients with PROC (Table 1). In a phase Ia study of patients with refractory solid tumors, 64% of patients (7/11) with heavily pretreated PROC treated with navicixizumab monotherapy achieved disease control.^203^ Besides, a phase Ib trial of navicixizumab plus paclitaxel in PROC was reported. Navicixizumab plus paclitaxel showed a promising clinical response with an ORR of 43.2% (95% CI, 28.3–59.0) and a DCR of 77.3% (95% CI, 62.2–88.5) in 44 enrolled patients with PROC.^204^ Another phase Ⅲ clinical trial (NCT05043402) has been registered to evaluate the efficacy of navicixizumab with or without paclitaxel in the treatment of PROC. Chun-Yan Lan et al purposely selected the combination of the oral agents apatinib and etoposide for investigation, and the results indicated that the combination of apatinib with etoposide displayed effective activity and manageable toxicity in patients with PROC or platinum-refractory ovarian cancer. The ORRs were achieved in 19 of 35 patients (54%; 95% CI, 36.6–71.2) in the intention-to-treat population. Of the 31 patients who had received at least one post-baseline efficacy assessment, 29 (94%) showed signs of tumor shrinkage.^205^ A randomized controlled phase Ⅲ trial to further validate these findings is ongoing (NCT04000295). Furthermore, the results of phase Ⅱ trials suggested that the combination of cedirandib and olaparib showed clinical activity in the treatment of PROC, especially in terms of enhancing mPFS improvement.^206^, ^207^, ^208^ The combination of cediranib and olaparib is important for platinum-free maintenance therapy in PROC. However, whether PROC patients can truly benefit from combination therapy needs to be further validated in phase Ⅲ trials (NCT02502266). Olvimulogen nanivacirepvec (Olvi-Vec) is a genetically engineered adenovirus that selectively infects and destroys cancer cells and simultaneously activates the body's immune system. In a nonrandomized phase Ⅱ clinical trial, Olvi-Vec followed by platinum-based chemotherapy with or without bevacizumab as immunochemotherapy demonstrated promising ORR (54%) and PFS (11.0 months) with a manageable toxicity profile in PROC.^209^ The phase Ⅲ trial (NCT05281471) of Olvi-Vec followed by platinum chemotherapy and bevacizumab in PROC is expected to be completed in 2024, with primary endpoint results presented in 2025.^210^ It's worth mentioning that the results of the ROSELLA phase Ⅲ trial, which aims to compare the efficacy of nab-paclitaxel with or without relacorilant (a selective glucocorticoid receptor antagonist) in patients with PROC were reported.^211^ In this study, 381 patients with PROC were randomly assigned to the combination group or the monotherapy group. Compared with those receiving nab-paclitaxel monotherapy, patients receiving the combination treatment showed a significant improvement both in mPFS (HR = 0.70; 95% CI: 0.54–0.91; log-rank *p* = 0.0076) and OS (HR = 0.69; 95% CI: 0.52–0.92; log-rank *p* = 0.0121), with similar adverse events. In the combination group, the mPFS and OS were 6.54 months (95% CI: 5.55–7.43) and 15.97 months (95% CI: 13.47‒not reached), respectively. In the nab-paclitaxel monotherapy group, the mPFS and OS were 5.52 months (95% CI: 0.94–5.88) and 11.50 months (95% CI: 10.02–13.57), respectively. The results significantly advanced the combination of relacorilant and nab-paclitaxel as a potential therapeutic strategy for treating patients with PROC.

**Table 1:** Ongoing phase Ⅲ clinical trials in platinum-resistant ovarian cancer.

Study	Drug/Treatment	Target	Control arm	Status
NCT06394492	SHR-A1921	TROP-2 antibody	IC chemotherapy	Not yet recruiting
NCT05622890	Mirvetuximab Soravtansine	FRα antibody		Recruiting
NCT05870748	Luveltamab tazevibulin	FRα antibody	IC chemotherapy	Recruiting
NCT06161025	Raludotatug Deruxtecan	CDH6 antibody	IC chemotherapy	Recruiting
NCT04921527	Chiauranib + Paclitaxel	Angiogenesis-related kinases inhibitor	Paclitaxel	Recruiting
NCT05043402	Navicixizumab ± Paclitaxel	Anti-VEGF and DLL4 antibody	Paclitaxel	Not yet recruiting
NCT04000295	Apatinib + Etopside	VEGFR2 inhibitor; topoisomerase inhibitor	Paclitaxel	Unknown
NCT04908787	BD0801 + IC chemotherapy	VEGFR inhibitor	IC chemotherapy	Active, not recruiting
NCT02502266	Cediranib + Olaparib	VEGFR inhibitor; PARP inhibitor	carboplatin	Active, not recruiting
NCT05145218	TQB2450+Anlotinib hydrochloride	PD-1 antibody; VEGFR inhibitor	Paclitaxel	Recruiting
NCT05092360	Nemvaleukin and/or pembrolizumab	IL2 receptor binder; PD-1 inhibitor	IC chemotherapy	Active, not recruiting
NCT05116189	Pembrolizumab + Paclitaxel ± Bev	PD-1 inhibitor	Paclitaxel ± Bev	Active, not recruiting
NCT04729387	Alpelisib + Olaparib	PI3K inhibitor; PARP inhibitor	Paclitaxel/PLD	Active, not recruiting
NCT00057720	TLK286	Glutathione analog	Topotecan/PLD	Completed
NCT00102973	TLK286+Carboplatin	Glutathione analog	PLD	Completed
NCT05257408	Relacorilant + Nab-Paclitaxel	GR modulator	Nab-paclitaxel	Completed
NCT03940196	NovoTTF-100 L(O) + Paclitaxel	–	Paclitaxel	Completed
NCT05316181	HIPEC + IC chemotherapy	–	IC chemotherapy	Recruiting
NCT05281471	Olvi-Vec + Platinum + Bev	–	IC chemotherapy + Bev	Completed

Note: CRS: cytoreduction surgery; HIPEC: hyperthermic intraperitoneal chemotherapy; Bev: bevacizumab; PLD: pegylated liposomal doxorubicin; PCC: physician-choice chemotherapy; IC: investigator's choice. GR: glucocorticoid receptor.

### Other feasible treatments

Given the low response rates and limited benefit of available therapeutics, effective treatments for PROC remain an urgent unmet need. In our opinion, several feasible approaches should be considered and implemented to overcome platinum resistance: 1) WEE1 inhibitors. As mentioned above, CCNE1 amplification is prevalent in primary PROC and WEE1 kinase inhibitors show a manageable toxicity profile and encouraging efficacy in epithelial ovarian cancer harboring CCNE1 amplification.^23^ Interestingly, the combination of WEE1 inhibitors with chemotherapy showed significant clinical activity in PROC.^212^ Furthermore, the WEE1 inhibitor adavosertib plus gemcitabine improved both PFS (HR: 0.55; 95% CI: 0.35–0.90; *p* = 0.015) and OS (HR: 0.56; 95% CI: 0.35–0.91; *p* = 0.017) compared to gemcitabine alone in PROC.^213^ 2) Emerging platinum (IV) prodrug nanotherapeutics. As we all know, reduced intracellular platinum concentration, caused by reduced uptake by transporters, is responsible for platinum resistance. Due to the lower toxicity and easier penetration of the cell membrane barrier, nanoscale drug delivery platforms (nDDPs) loaded with platinum analogues as payloads could be a possible approach to solve the problem in the treatment of PROC.^214^, ^215^, ^216^ 3) RNAi-based therapeutics. To date, few RNAi-based drugs have entered clinical trials and even been approved by the FDA, indicating that RNAi-based therapeutics is becoming more and more sophisticated.^217^^,^^218^ RNAi-based therapeutics may represent great potential for the development of targeted therapies for PROC.^219^ With the development of nucleic acid delivery systems, we may even be able to achieve personalized medicine by combining it with RNA sequencing. 4) Combination of sodium arsenite (NaAsO_2_) and hyperthermic intraperitoneal chemotherapy (HIPEC). It has been reported that NaAsO_2_ and hyperthermia sensitize tumors to cisplatin by inhibiting NER and increasing tumor platinum uptake *in vivo*.^220^ 5) PI3K inhibitors. Numerous studies have demonstrated that abnormal activation of the PI3K/Akt/mTOR signaling pathway leads to platinum resistance.^221^ Alpelisib, an orally bioavailable, small-molecule α-specific PI3K inhibitor, has been approved to treat advanced breast cancer patients who are PIK3A-mutated, HR-positive, and HER2-negative. Importantly, PI3KCA mutations or gene amplification are prevalent with 30.5% of all ovarian cancers and 45% of the endometrioid and clear cell subtypes.^222^ Therefore, alpelisib could be a good choice for patients with PROC either as a standalone intervention or in combination with other chemotherapies. 6) TLK286/Buthionine Sulfoximine. PROC cells are enriched with intracellular GSH, and deprivation of GSH promotes the chemotherapy response through ROS accumulation, detoxification reduction, and ferroptosis. TLK286 is a novel glutathione analogue activated by glutathione S-transferase P1-1. A phase Ⅱ study revealed that TLK286 in combination with PLD is well tolerated and effective in PROC.^223^ In addition, buthionine sulfoximine (BSO), a novel inhibitor of GSH synthesis, may also be a potential drug to treat PROC.^224^^,^^225^

## Conclusions and perspectives

Despite the exploration of platinum-resistant mechanisms and the deepening of clinical research, PROC patients still show extremely poor prognosis. Overcoming platinum resistance and managing PROC patients in clinic requires addressing the following issues. Firstly, patients with nominal PROC may still respond well to platinum-based chemotherapy. Although the NCCN ovarian cancer guidelines differentiate platinum-sensitive ovarian cancer from PROC.^226^ However, the definition of PROC is imprecise. In particular, the implementation of PARPi and bevacizumab adds complexity in interpreting platinum resistance.^227^ Accurate identification of PROC patients has become a challenge in the management of PROC patients. Secondly, although multiple potential mechanisms of platinum resistance have been discovered, the contribution weights of these mechanisms to PROC are incomprehensible. Due to the heterogeneity of OC, distinguishing the cause of platinum resistance is crucial for selecting effective treatment strategies. Thirdly, there are challenges in sample collection following platinum-based chemotherapy. Samples collected at initial surgery have been widely used in studies of PROC, but these samples collected at initial surgery cannot accurately reflect the genomic characteristics or gene expression patterns of patients at the time of platinum resistance. Fourthly, current models and platforms for investigating PROC, such as patient-derived xenografts (PDXs) and cell-derived xenografts (CDXs), face various problems. For instance, PDXs grossly retain tumor heterogeneity, and the clinical response to platinum-based chemotherapy is limited.^228^ Nevertheless, the human microenvironment is gradually substituted by the murine stroma and vasculature, and immunocompromised mice never fully recapitulate the complex crosstalk between the human immune system and the tumor.^229^, ^230^, ^231^

**Figure 4:**
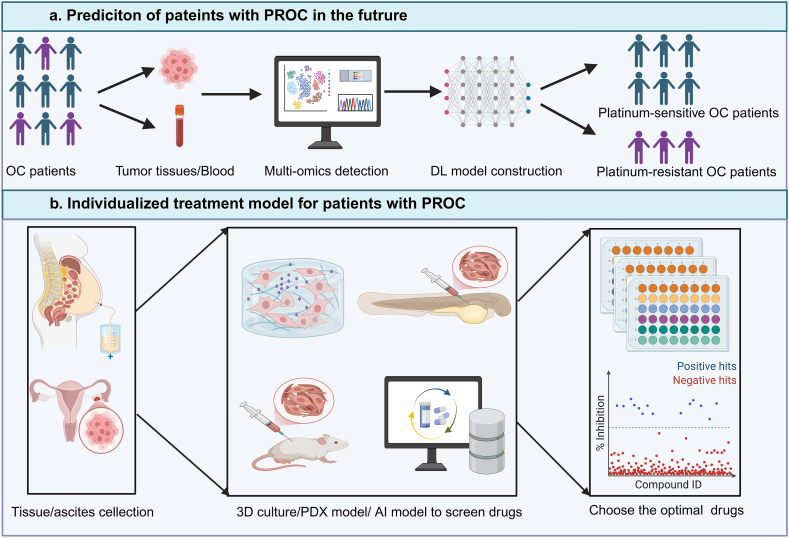
The prediction and individualized treatment model of patients with PROC in the future.

Considering the development of multi-omics, and the increasing popularity of personalized treatment, we proposed a novel individualized identification and treatment model for patients with PROC (Fig. 4). Obviously, accurate prediction of the platinum response is crucial for strategically selecting postoperative interventions to mitigate the risks associated with suboptimal therapeutic outcomes and adverse effects.^232^ In our opinion, we recommend the integration of multi-omics analysis, including proteomic sequencing, single-cell sequencing, bulk RNA sequencing, iconography, and histopathology, to construct a multi-modal model to accurately predict the initial platinum-based chemotherapy response in OC patients. This could make the therapeutic decision easier, and patients benefit more from the clinical outcome. At present, several promising predictive models have been reported. For instance, Shen et al constructed a prediction model by using three immune-related proteins, CCR1, IGHV_35 and CD72, in tissue-derived extracellular vesicles, with one clinical parameter, the presence of postoperative residual lesions. The predictive model has a highly accurate performance, with the area under the curve (AUC) values of 0.960 and 0.864 for the training and testing sets, respectively.^233^ Wanger and his colleagues established a model with high performance by using HE4, CA125, TMEM205, and CFH within extracellular vesicle.^234^ Besides, several multimodal deep learning models based on histopathological images or radiomic data have been developed to predict platinum response.^235^, ^236^, ^237^ However, all these predictive models were built and validated with small samples. Whether they can be used to predict platinum-based treatment responses of patients with OC in clinic requires further testing with a larger sample size. Once platinum resistance develops, it becomes crucial to promptly identify the most effective therapeutic agent, given the limited survival time. Currently, PDX models established in mice or zebrafish have emerged as valuable tools for screening optimal therapeutic agents. Compared to the mouse PDX model, the zebrafish PDX model not only effectively mirrors the patient's response to treatment agents but also has several advantages, including higher success rates and shorter construction times.^238^ Considering the limited survival time of PROC patients, especially the primary PROC patients, the zebrafish PDX model might be a better option. Due to the clinical superiority of 3D culture, 3D models have the potential to better predict patient-specific responses to treatment. Recently, a novel 3D high-throughput phenotypic drug screening pipeline was established using patient-derived polyclonal spheroids isolated from ascites fluid.^239^ The 3D spheroid model can represent good surrogates for clinical response. It is worth mentioning that it has expedited the identification of new promising candidates and offered the potential of drug repurposing to rapidly advance new treatments into clinical trials. Chemotherapeutic assay (ChemoID) is a functional precision medicine assay that uses an individual patient's live bulk of tumor cells and CSCs isolated from tumor biopsies or malignant fluid aspirates (peritoneal and/or pleural fluid) to help physicians select appropriate chemotherapies for individual patients. Recent studies demonstrated that ChemoID-guided therapy improved the ORR, DOR, and PFS compared to the physician's choice of chemotherapy.^240^ In conclusion, significant progress has been made in the identification and treatment of PROC patients in recent years. However, there is still a long way to go before individualized treatment can be implemented for patients with PROC. More preclinical studies and clinical trials, especially trials of biomarker-guided therapies, should be designed and exploited.

## CRediT authorship contribution statement

**He Li:** Writing – review & editing, Writing – original draft, Funding acquisition. **Jia-Jia Sheng:** Writing – review & editing, Visualization, Writing – original draft. **Sheng-An Zheng:** Visualization, Conceptualization, Writing – review & editing. **Po-Wu Liu:** Writing – review & editing, Visualization. **Nayiyuan Wu:** Writing – review & editing, Visualization. **Wen-Jing Zeng:** Writing – review & editing, Investigation, Methodology, Conceptualization. **Ying-Hua Li:** Conceptualization, Investigation, Methodology, Writing – review & editing. **Jing Wang:** Investigation, Methodology, Conceptualization, Writing – review & editing.

## Funding

This research was supported by the Young Scientists Fund of the 10.13039/501100001809National Natural Science Foundation of China (No. 82303035) and the 10.13039/501100004735Natural Science Foundation of Hunan Province, China (No. 2024JJ5245).

## Conflict of interests

The authors declare that there are no conflict of interests.
